# Horizon Europe: the DELIVER (DELiberative ImproVEment of oRal care quality) project

**DOI:** 10.1093/eurpub/ckac129.373

**Published:** 2022-10-25

**Authors:** P Vassallo

**Affiliations:** 1 Radboud Institute for Health Science, Radboud University, Nijmegen, Netherlands; 2 Poliklinik für Zahnerhaltungskunde, Heidelberg University Hospital, Heidelberg, Germany

## Abstract

Oral diseases and conditions affect more than 3.5 billion people worldwide. They are the 3rd most expensive diseases to treat in the EU and disproportionally affect vulnerable groups. In deviation from the UN and WHO goal of Universal Health Coverage, many EU citizens do not have access to quality oral care without financial hardship. To this end, the DELIVER (DELiberative ImproVEment of oRal care quality) project aims to enhance the quality of oral care through deliberative dialogue and action involving citizens, patients, providers, payers and policymakers. DELIVER will create a synergistic problem-solving ecosystem to convert deliberative dialogues into meaningful improvement of oral care quality. Using a mixed-methods research approach, DELIVER will co-develop and co-produce new quality improvement approaches in three phases. The 1st phase involves situational analysis, consenting of core quality indicators, and development of a EU-wide monitoring framework. The 2nd phase involves in-depth analysis of select quality improvement approaches: (i) PROMs/PREMs-based quality improvement in dental practices; (ii) community-based quality improvement for vulnerable groups; (iii) quality-oriented commissioning of oral health services. The regulatory determinants of oral care quality improvement will be scrutinized. In the 3rd and final phase, the knowledge gained in the 1st and 2nd phases will be merged into the DELIVER Quality Toolkit with manuals and digital tools for concretely actionable and context-adaptive approaches for oral care quality improvement. This presentation will give an overview of the DELIVER project and discuss how it can contribute to improving oral health systems.

